# Clinical and economic outcomes with rivaroxaban versus warfarin in patients with nonvalvular atrial fibrillation and obstructive sleep apnea: retrospective analysis of US healthcare claims

**DOI:** 10.1007/s10840-024-01940-6

**Published:** 2024-11-25

**Authors:** Andrea Natale, Sanghamitra Mohanty, Cindy Chen, Yuan Zhao, Alicia K. Campbell, Brahim Bookhart, Veronica Ashton

**Affiliations:** 1 Texas Cardiac Arrhythmia Institute, St. David’s Medical Center, Austin, TX USA; 2 Interventional Electrophysiology, Scripps Clinic, San Diego, CA USA; 3 Metro Health Medical Center, Case Western Reserve University School of Medicine, Cleveland, OH USA; 4 Department of Biomedicine and Prevention, Division of Cardiology, University of Tor Vergata, Rome, Italy; 5 Janssen Scientific Affairs, LLC, a Johnson & Johnson Company, Titusville, NJ USA

**Keywords:** Atrial fibrillation, Healthcare resource utilization, Rivaroxaban, Sleep apnea

## Abstract

**Background:**

Atrial fibrillation (AF) and obstructive sleep apnea (OSA) are often comorbid and associated with increased risk of cardiovascular events such as stroke. We evaluated the effectiveness, safety, healthcare resource utilization, and costs of rivaroxaban versus warfarin in patients with nonvalvular AF (NVAF) and comorbid OSA.

**Methods:**

We used the IQVIA PharMetrics^®^ Plus adjudicated claims database to evaluate patients with NVAF, OSA, and moderate-to-severe stroke risk who initiated rivaroxaban or warfarin between November 2011 and December 2022. We adjusted for potential confounders with propensity score overlap weighting. Primary endpoints were evaluated based on intent-to-treat (ITT) and on-treatment follow-up to compare stroke or systemic embolism risk, major bleeding risk, all-cause healthcare resource utilization (inpatient hospitalizations, emergency department visits, outpatient visits, and pharmacy fills), and costs (per patient per year [PPPY]) by treatment cohort.

**Results:**

In total, 14,765 patients were included (9133 received rivaroxaban; 5632 received warfarin). Rivaroxaban significantly reduced stroke or systemic embolism versus warfarin by 26% (ITT—hazard ratio, 0.74 [95% CI 0.60–0.91]; *P* = 0.004) and 30% (on-treatment—hazard ratio, 0.70 [95% CI 0.55–0.89]; *P* = 0.004). Major bleeding was not significantly different between rivaroxaban and warfarin in either analysis. All-cause healthcare resource utilization was significantly reduced with rivaroxaban versus warfarin, leading to significantly reduced PPPY costs.

**Conclusions:**

Among patients with NVAF and OSA, rivaroxaban was associated with a significant reduction in stroke or systemic embolism risk versus warfarin with no difference in major bleeding. Rivaroxaban significantly reduced healthcare resource utilization and costs compared with warfarin, providing support for the use of rivaroxaban in this population.

**Supplementary Information:**

The online version contains supplementary material available at 10.1007/s10840-024-01940-6.

## Introduction

In the USA, up to 6 million people have atrial fibrillation (AF) and, by 2050, AF is expected to affect up to 16 million people [1]. AF is associated with increased morbidity and mortality, primarily due to cardiovascular comorbidities, including stroke and venous thromboembolism [1, 2]. Common risk factors for AF and cardiovascular disease include aging, obesity, hypertension, diabetes mellitus, and obstructive sleep apnea (OSA) [1, 3–7]. Approximately 25% of adults in the USA have OSA, and medical claims for sleep apnea have increased by 850% from 2014 to 2017 [6, 8]. AF and OSA share cardiovascular and metabolic risk factors; obesity is among the most important risk factors for OSA that contributes to cardiovascular dysfunction and abnormalities associated with metabolic syndrome [6, 9]. AF and OSA also have linked pathophysiology, including structural remodeling, neurohormonal activation, and coagulation abnormalities [4, 5, 10, 11]. Among patients with AF, the estimated prevalence of OSA ranges from 18 to 85% [5, 12, 13]. The Heart Rhythm Society reported that approximately 50% of patients with AF have sleep apnea, and patients with sleep apnea have a four-fold increased risk of developing AF [14]. Patients who have AF and comorbid OSA are more likely to receive anticoagulation therapy for stroke prevention [12]. Oral anticoagulants, including rivaroxaban and warfarin, are recommended by guidelines as first-line treatment to prevent stroke and systemic embolism for patients with nonvalvular AF (NVAF) who have moderate-to-high stroke risk, as defined by a CHA_2_DS_2_-VASc score ≥ 2 in men or ≥ 3 in women [3, 7]. Rivaroxaban, a factor Xa inhibitor, also has been shown to significantly attenuate cardiac remodeling due to intermittent hypoxia through the prevention of oxidative stress and fibrosis in mice [15]. These findings suggest that treatment with rivaroxaban may provide therapeutic benefits in patients with NVAF and comorbid OSA. Here, we evaluated the effectiveness, safety, healthcare resource utilization (HCRU), and costs among patients with NVAF with OSA who initiated treatment with rivaroxaban compared with warfarin in a US healthcare claims database.

## Methods

### Study design

This retrospective cohort study was conducted between November 1, 2010, and December 23, 2022. Evaluable patients with NVAF and OSA initiating rivaroxaban or warfarin were identified between November 1, 2011, and December 23, 2022 (Online Resource 1; Supplementary Fig. 1). The primary objectives were to compare the incidence of stroke or systemic embolism and major bleeding and compare all-cause HCRU and costs during the follow-up period among patients who initiated rivaroxaban versus warfarin. Secondary objectives included a comparison of the risks of ischemic stroke, intracranial hemorrhage, systemic embolism, and major extracranial bleeds with rivaroxaban versus warfarin. Exploratory objectives were to compare the effectiveness and safety of rivaroxaban versus warfarin in subgroups of patients with NVAF and OSA by age, sex, presence of obesity, diabetes, heart failure, prior stroke, presence of metabolic syndrome (defined as the presence of various metabolic abnormalities, including obesity, hypertension, hyperlipidemia, and type 2 diabetes mellitus [9]), and use of positive airway pressure (PAP) therapy. Adherence to rivaroxaban and warfarin was also evaluated.

### Data sources

We used the IQVIA PharMetrics^®^ Plus adjudicated health plan claims database, which includes information from > 70 contributing health plans and self-insured employer groups throughout the USA covering > 140 million unique individuals since 2006. This database includes medical and pharmacy claims data (costs and descriptive services), patient-level enrollment records, and patient demographics. This database is generally representative of commercially insured individuals < 65 years of age, with 6% having Medicare, 3% having Medicaid, and 1% having other insurance. The mean length of enrollment is ≥ 39 months, and ≥ 47 million patients have ≥ 3 years of continuous enrollment for medical and pharmacy coverage.

### Patients

Patients newly initiating rivaroxaban or warfarin were identified. Eligible patients had ≥ 1 pharmacy claim for rivaroxaban or warfarin between November 1, 2011, and December 23, 2022, with the date of the first claim being defined as the index date; ≥ 1 medical claim with a diagnosis code for OSA (*International Classification of Diseases, 9th/10th Revision, Clinical Modification* [ICD-9-CM/ICD-10-CM] codes—327.20, 327.23, 327.29, 780.51, 780.53, 780.57, G47.30, G47.33, and G47.39) in any diagnostic position during the baseline period or on the index date; and ≥ 1 diagnosis of AF (ICD-9-CM/ICD-10-CM codes—427.31, I48.0x–I48.2x, I48.91x) during the baseline period or on the index date. Included patients were aged ≥ 18 years on the index date, had a moderate-to-high risk of stroke (CHA_2_DS_2_-VASc score ≥ 2 in men and ≥ 3 in women) per 2019 American Heart Association guidelines [3], and had ≥ 365 days of continuous health plan enrollment prior to the index date. Patients were excluded if they had ≥ 1 of the following: diagnosis code for valvular AF (e.g., presence of mitral stenosis or prior heart valve replacement) at any time during the baseline period, diagnosis code for other indications for oral anticoagulant use (e.g., venous thromboembolism, prophylaxis after knee or hip replacement), claim for an oral anticoagulant during the baseline period, or claim for pregnancy during the baseline period or on the index date. After checking the patients’ profiles, we restricted the analysis to those with an index date on or after 2013 to allow time for a recently approved medication to be prescribed and because early adopters may have different characteristics.

### Outcomes

Clinical (effectiveness and safety) and economic (HCRU and costs) outcomes were evaluated using 2 follow-up approaches. The intent-to-treat (ITT) approach evaluated the overall risk of outcomes after initiating treatment. Patients were followed from the index date until the earliest of the following: first clinical outcome event, health plan disenrollment, or last data point. The on-treatment approach evaluated outcomes during the period in which patients were exposed to treatment. Patients were followed from the index date until the earliest of the first clinical outcome event, health plan disenrollment, last data point, or discontinuation of index treatment (defined as a gap of > 60 days between 2 prescription fills of index treatment or a treatment change to another oral anticoagulant).

The primary effectiveness outcome was the incidence (per 100 person-years of follow-up) of stroke or systemic embolism during the follow-up period. Stroke or systemic embolism was defined as ≥ 1 hospitalization with a primary diagnosis of stroke (ischemic or hemorrhagic) or systemic embolism (ICD-9-CM—430.x–432.x, 433.01, 433.11, 433.21, 433.31, 433.81, 433.91, 434.01, 434.11, 434.91, 436.x, 444.01, 444.09, 444.1, 444.21, 444.22, 444.81, 444.89, 444.9; ICD-10-CM—I60.x–I62.x, I63.x, I74.x). The primary safety outcome was the incidence (per 100 person-years of follow-up) of major bleeding after the index date, evaluated using a validated claims-based algorithm [16]. All-cause HCRU and costs incurred during the follow-up period were compared between treatments. HCRU outcomes included inpatient hospitalizations and length of stay (days), emergency department (ED) visits, outpatient visits (hospital outpatient, physician office [primary and specialty care], and skilled nursing facility visits), and prescription fills. All costs were inflated to 2022 US dollars and presented as mean costs per patient per year (PPPY) for total healthcare costs, which included the total medical and pharmacy costs. Medical costs included inpatient hospitalizations, ED visits, and outpatient visits. Pharmacy costs included only all-cause pharmacy expenses.

Secondary outcomes were the incidence (per 100 person-years of follow-up) of ischemic stroke, intracranial hemorrhage, and major extracranial bleeding, evaluated as separate outcomes. Ischemic stroke and intracranial hemorrhage were defined as ≥ 1 hospitalization with a primary diagnosis code of ischemic stroke or intracranial hemorrhage, respectively. Major extracranial bleeding included all major bleeding events from the Cunningham algorithm that did not occur in the head [16].

Exploratory objectives included comparisons of effectiveness and safety stratified by subgroups based on baseline characteristics of age (≥ 65 vs. < 65 years), sex, presence/absence of obesity (ICD-9-CM codes for body mass index ≥ 30 kg/m^2^), diabetes, heart failure, prior stroke, presence/absence of metabolic syndrome, and use of PAP therapy. Adherence to rivaroxaban and warfarin was measured by the proportion of days covered.

### Statistical analysis

All patients within the database satisfying the eligibility criteria were evaluated. To adjust for potential confounding between rivaroxaban and warfarin cohorts, a multivariable logistic regression model [17] was used to calculate propensity scores, considering demographics (age, sex, race, insurance type, US census region, type of AF diagnosis) and baseline characteristics (comorbidities, continuous or bilevel PAP, sleep study, surgical treatment of OSA, intracranial hemorrhage, stroke or systemic embolism, transient ischemic attack, major bleeding, prior cardiovascular procedures, number of hospitalizations during baseline, CHA_2_DS_2_-VASc score, HAS-BLED score, and concomitant medications). Estimated propensity scores were used to weight patients using an overlap weighting approach [18], which assigns weights to patients that are proportional to their probability of belonging to the opposite cohort and adjusts for confounders in all eligible patients. This allows all eligible patients to remain in the analysis, resulting in an exact balance between the cohorts. Sufficient overlap of the propensity score between the cohorts was assessed through the calculation of equipoise.

Demographic and baseline characteristics for each cohort were summarized using descriptive statistics (categorical variables as percentages; continuous variables as means and SDs). Time-to-first-event outcomes were assessed using propensity score overlap weighted Cox proportional hazards regression models using a robust estimator to calculate hazard ratios and corresponding 95% CIs. All-cause HCRU was reported as PPPY, calculated as the number of events divided by total person-years of follow-up. Direct healthcare costs were reported as mean costs PPPY, calculated as the cost incurred during follow-up divided by the total person-years during follow-up. Between-group differences in HCRU and costs were estimated using a Poisson regression model with a log link (or negative-binomial models where appropriate to account for overdispersion). For exploratory subgroup analyses, propensity score models and weighting were rerun for each subgroup including the same variables as the primary analysis. To assess adherence, the proportion of days covered was calculated as the ratio of the number of days covered by the index medication prescription dispensed during the follow-up period divided by the number of days of follow-up. A proportion of days covered threshold ≥ 0.8 was considered adherent, and < 0.8 was considered nonadherent. The proportions of patients adherent to each treatment were compared with an odds ratio and 95% CIs.

All database management and statistical analysis were performed using SAS version 9.4 (SAS Institute, Cary, NC, USA). A *P*-value < 0.05 was considered statistically significant.

## Results

### Patient characteristics

Of 181,337 patients with NVAF, continuous health plan enrollment for ≥ 12 months, and a prescription for rivaroxaban or warfarin, 41,374 (22.8%) had a concurrent diagnosis code for OSA (Online Resource 1; Supplementary Fig. 2). After applying the remaining eligibility criteria, 14,765 patients were included: 9133 received rivaroxaban, and 5632 received warfarin. Overall, the mean (SD) follow-up time was 2.30 (2.15) years (rivaroxaban 2.31 [2.12], warfarin 2.29 [2.19]). Demographic and baseline characteristics of the treatment cohorts were well balanced after propensity score overlap weighting (Table 1). The mean age was 63 years, and the mean CHA_2_DS_2_-VASc and HAS-BLED scores were 3.5 and 2.6, respectively, in both cohorts. Only 0.2% of patients had sleep apnea testing or treatment (i.e., composite of PAP, home sleep apnea testing, sleep study, and surgical treatment of OSA) at baseline. The most common comorbidities (i.e., those occurring in > 50% of patients in both cohorts) were hypertension (94.5%), hyperlipidemia (76.5%), obesity (57.3%), and diabetes (58.6%). P2Y12 inhibitors, aspirin, and other antiplatelet drugs were used by 14.3%, 4.4%, and 1.5%, respectively, in both cohorts.

**Table 1:** Baseline demographic and characteristics before and after PS overlap weighting

Characteristic	Before weighting			After weighting		
	Rivaroxaban (n = 9133)	Warfarin (n = 5632)	Standardized difference^a^	Rivaroxaban (n = 9133)	Warfarin (n = 5632)	Standardized difference^a^
Age, years, mean (SD)	62.3 (8.9)	63.8 (9.0)	16.8%	63.3 (5.0)	63.4 (6.3)	2.3%
Sex (%)						
Male	76.1%	73.0%	7.2%	73.9%	73.9%	0.0%
Female	23.9%	27.0%	7.2%	26.1%	26.1%	0.0%
Type of index AF diagnosis (%)						
Paroxysmal	41.8%	26.9%	31.9%	31.4%	31.4%	0.0%
Persistent	4.5%	3.7%	4.3%	4.0%	4.0%	0.0%
Chronic	2.4%	3.1%	3.8%	2.9%	2.9%	0.0%
Unspecified	51.2%	66.4%	31.2%	14.3%	14.3%	0.0%
Insurance type (%)						
Medicare^b^	18.3%	27.1%	21.2%	23.7%	23.7%	0.0%
Commercial	53.7%	45.8%	15.7%	49.4%	49.4%	0.0%
Medicaid	1.6%	5.0%	19.2%	3.2%	3.2%	0.0%
Self-insured	26.3%	21.8%	10.5%	23.4%	23.4%	0.0%
Prescription only	0.0%	0.0%	2.1%	0.0%	0.0%	0.0%
Unknown/missing	0.2%	0.3%	2.6%	0.3%	0.3%	0.0%
US census region (%)						
Northeast	17.7%	20.0%	5.8%	19.4%	19.4%	0.0%
Midwest	30.0%	36.2%	13.1%	34.1%	34.1%	0.0%
South	37.4%	24.5%	28.3%	28.5%	28.5%	0.0%
West	14.0%	18.5%	12.2%	17.1%	17.1%	0.0%
Baseline risk scores, mean (SD)						
CHA_2_DS_2_-VASc	3.2 (1.3)	3.7 (1.5)	38.7%	3.5 (0.8)	3.5 (0.9)	0.0%
HAS-BLED	2.4 (1.1)	2.8 (1.2)	27.6%	2.6 (0.7)	2.6 (0.8)	1.4%
Baseline comorbidities^c^ (%)						
Hypertension	94.6%	94.8%	0.6%	94.5%	94.5%	0.0%
Hyperlipidemia	76.5%	77.1%	1.3%	76.5%	76.5%	0.0%
Diabetes	55.8%	62.1%	10.4%	58.6%	58.6%	0.0%
Obesity	58.9%	57.5%	2.3%	57.3%	57.3%	0.0%
Heart failure	41.3%	55.1%	22.9%	48.1%	48.1%	0.0%
Chronic kidney disease	31.1%	45.0%	24.0%	36.2%	36.2%	0.0%
Ischemic (coronary) heart disease	26.3%	42.9%	29.4%	34.9%	34.9%	0.0%
Osteoarthritis	31.7%	32.2%	0.8%	32.3%	32.3%	0.0%
Chronic obstructive pulmonary disease	21.7%	31.1%	17.9%	26.6%	26.6%	0.0%
Gastroesophageal reflux disease/heartburn	26.4%	24.9%	2.7%	25.0%	25.0%	0.0%
Depression	18.0%	21.1%	6.4%	19.7%	19.7%	0.0%
Hypothyroidism	15.8%	18.9%	6.9%	17.6%	17.6%	0.0%
Myocardial infarction	13.8%	20.6%	15.3%	16.4%	16.4%	0.0%
Acute kidney injury	11.2%	24.4%	30.2%	16.4%	16.4%	0.0%
Anemia	13.6%	22.1%	18.8%	16.3%	16.3%	0.0%
Asthma	14.8%	17.1%	5.0%	16.3%	16.3%	0.0%
Anxiety	16.7%	16.7%	0.0%	16.2%	16.2%	0.0%
Peripheral vascular attack	13.4%	19.7%	14.4%	15.9%	15.9%	0.0%
Pneumonia	11.5%	19.0%	17.8%	14.6%	14.6%	0.0%
Acute coronary syndrome	10.6%	18.2%	18.7%	14.0%	14.0%	0.0%
Smoker	14.1%	13.6%	1.2%	13.5%	13.5%	0.0%
Diverticulitis	10.4%	10.4%	0.1%	10.1%	10.1%	0.0%
Solid tumor	8.6%	9.6%	2.9%	9.1%	9.1%	0.0%
Osteoporosis	7.0%	9.7%	8.4%	8.7%	8.7%	0.0%
Ischemic stroke < 30 days prior to index date	5.8%	10.5%	14.9%	8.2%	8.2%	0.0%
Coagulopathy	4.6%	10.7%	20.2%	6.9%	6.9%	0.0%
Chronic venous insufficiency	5.7%	8.1%	8.1%	6.7%	6.7%	0.0%
Transient ischemic attack	6.2%	7.0%	2.8%	6.6%	6.6%	0.0%
Hemorrhoids	6.3%	5.7%	2.1%	6.0%	6.0%	0.0%
Rheumatoid arthritis/collagen vascular disease	5.8%	6.1%	0.9%	6.0%	6.0%	0.0%
Liver disease	4.5%	7.3%	10.1%	5.8%	5.8%	0.0%
Carotid stenosis	5.6%	6.1%	1.7%	5.6%	5.6%	0.0%
Cardioversion	3.5%	7.2%	14.4%	4.9%	4.9%	0.0%
Proteinuria	3.1%	6.0%	11.8%	4.3%	4.3%	0.0%
Varicose veins	3.6%	4.8%	4.8%	4.2%	4.2%	0.0%
Any prior major bleeding^d^	2.7%	6.4%	15.7%	4.2%	4.2%	0.0%
Aortic plaque	3.7%	5.0%	5.2%	4.1%	4.1%	0.0%
Coronary artery bypass grafting	2.1%	6.7%	20.5%	3.8%	3.8%	0.0%
Headache	3.5%	3.4%	0.6%	3.4%	3.4%	0.0%
Percutaneous coronary intervention	2.3%	4.7%	11.2%	3.3%	3.3%	0.0%
Alcohol abuse	2.3%	2.6%	1.7%	2.5%	2.5%	0.0%
Gastrointestinal bleeding^d^	1.3%	3.6%	13.1%	2.1%	2.1%	0.0%
Dementia	1.5%	2.6%	6.4%	2.0%	2.0%	0.0%
Psychosis	1.2%	2.5%	8.0%	1.8%	1.8%	0.0%
Ablation	1.5%	1.9%	2.9%	1.7%	1.7%	0.0%
Metastatic cancer	1.7%	1.6%	0.6%	1.6%	1.6%	0.0%
Major adverse limb events	1.1%	1.7%	4.7%	1.4%	1.4%	0.0%
End-stage renal disease	0.5%	7.3%	34.3%	1.4%	1.4%	0.0%
Lymphoma	1.0%	1.5%	4.3%	1.2%	1.2%	0.0%
Intracranial hemorrhage	0.7%	1.5%	6.4%	1.1%	1.1%	0.0%
Systemic embolism	0.7%	1.3%	5.4%	1.0%	1.0%	0.0%
Presence of sleep apnea testing/treatment^e^	0.2%	0.3%	1.8%	0.2%	0.2%	0.0%
Baseline medication use^c^ (%)						
Other antiarrhythmic drugs^f^	68.8%	67.9%	1.6%	67.6%	67.6%	0.0%
Beta-blockers	65.1%	65.4%	0.5%	64.6%	64.6%	0.0%
Statins	59.7%	60.3%	1.0%	59.7%	59.7%	0.0%
ACEIs or ARBs	58.8%	56.2%	4.4%	56.8%	56.8%	0.0%
Antidiabetic drugs	43.3%	45.9%	4.4%	43.8%	43.8%	0.0%
Loop diuretics	31.4%	42.1%	18.5%	36.7%	36.7%	0.0%
Proton pump inhibitors	28.5%	28.1%	0.7%	28.1%	28.1%	0.0%
Dihydropyridine calcium channel blockers	23.7%	25.4%	3.3%	23.7%	23.7%	0.0%
SSRIs or SNRIs	22.2%	24.0%	3.6%	23.4%	23.4%	0.0%
NSAIDs	25.0%	20.0%	9.8%	22.2%	22.2%	0.0%
Strong CYP3A4 inhibitors	20.2%	21.1%	1.8%	21.0%	21.0%	0.0%
Thiazide diuretics	17.8%	19.2%	2.8%	18.4%	18.4%	0.0%
P2Y12 inhibitors	12.5%	15.9%	8.1%	14.3%	14.3%	0.0%
Levothyroxine	11.8%	14.9%	7.5%	13.6%	13.6%	0.0%
Diltiazem	14.2%	12.0%	5.2%	12.9%	12.9%	0.0%
Benzodiazepines	13.4%	12.6%	1.9%	12.8%	12.8%	0.0%
Other cholesterol-lowering drugs	13.2%	12.5%	1.5%	12.5%	12.5%	0.0%
Other antidepressants	11.3%	13.0%	4.3%	12.1%	12.1%	0.0%
Amiodarone	7.8%	6.3%	4.8%	6.6%	6.6%	0.0%
Digoxin	5.2%	6.1%	3.1%	5.9%	5.9%	0.0%
Histamine-2 receptor antagonists	4.7%	5.6%	3.5%	5.0%	5.0%	0.0%
Aspirin	4.6%	5.0%	1.3%	4.4%	4.4%	0.0%
Alpha-blockers	3.9%	5.2%	4.9%	4.3%	4.3%	0.0%
Strong CYP3A4 inducers	3.4%	3.4%	0.1%	3.5%	3.5%	0.0%
Verapamil	2.1%	2.0%	0.5%	2.1%	2.1%	0.0%
Other antiplatelet drugs	1.4%	1.6%	1.2%	1.5%	1.5%	0.0%
All-cause healthcare resource utilization counts, mean (SD)						
Inpatient hospitalizations	5.8 (11.7)	12.2 (19.8)	39.1%	8.2 (9.0)	8.2 (9.0)	0.5%
ED visits	0.5 (1.3)	0.6 (1.4)	6.6%	0.6 (0.9)	0.5 (0.9)	2.5%
Outpatient visits	31.0 (24.5)	40.2 (38.2)	28.7%	33.21 (15.1)	34.6 (19.6)	7.7%
Prescription fills	26.8 (18.8)	28.0 (21.1)	5.8%	27.6 (10.9)	26.8 (14.3)	5.9%
Costs ($USD), mean (SD)						
Total medical	30,325 (47,982)	60,175 (109,326)	14.6%	39,252 (36,743)	39,252 (37,895)	0.0%
Inpatient hospitalization	17,707 (40,411)	41,276 (95,018)	32.3%	25,597 (32,225)	25,597 (32,255)	0.0%
ED visit	527 (1620)	558 (1779)	1.8%	517 (886)	517 (1209)	0.0%
Outpatient visit	12,091 (20,203)	18,341 (46,744)	17.4%	13,138 (12,658)	13,138 (16,812)	0.0%
Total pharmacy	5017 (10,466)	5303 (11,832)	2.6%	5085 (5500)	5085 (8787)	0.0%
Total (medical + pharmacy)	35,342 (49,962)	65,478 (111,263)	34.9%	44,337 (37,674)	44,337 (40,141)	0.0%

*ACEI* angiotensin-converting enzyme inhibitor, *ARB* angiotensin receptor blocker, *ED* emergency department, *NSAID* nonsteroidal anti-inflammatory drug, *OSA* obstructive sleep apnea, *PS* propensity score, *SD* standard deviation, *SSRI* selective serotonin reuptake inhibitor, *SNRI* serotonin-norepinephrine reuptake inhibitor, *USD* US dollar

^a^Standardized difference < 10% was considered a negligible imbalance

^b^Medicare includes Medicare Part C, Medicare Advantage, and Medicare supplemental plans

^c^The following clinical characteristics with a prevalence < 1% were not included in the propensity score model: lower extremity paralysis, Crohn’s or ulcerative colitis, *Helicobacter pylori*, orthopedic surgery, hypercoagulable state, and barbiturates

^d^Major and gastrointestinal bleeding were defined based on the Cunningham algorithm [16]

^e^Presence of sleep apnea testing/treatment is a composite of positive airway pressure use, home sleep apnea testing, sleep study, and surgical treatment of OSA

^f^Anti-arrhythmic drugs include sodium channel blockers (disopyramide, quinidine, flecainide), all beta-blockers, potassium channel blockers (amiodarone, dronedarone, sotalol), and all calcium channel blockers

### Effectiveness and safety outcomes

**Fig. 1:**
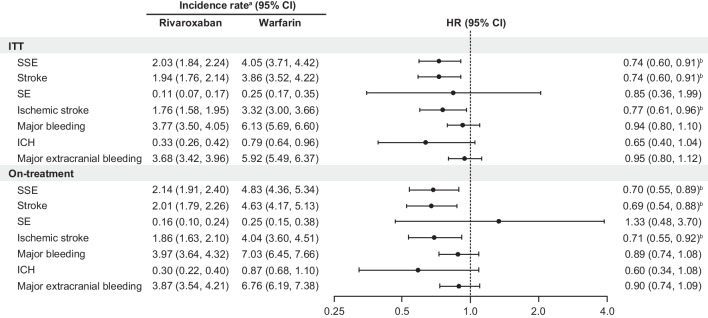
Clinical outcomes with rivaroxaban vs. warfarin in patients with NVAF and sleep apnea (PS overlap weighted). CI, confidence interval; HR, hazard ratio; ITT, intent-to-treat; NVAF, nonvalvular atrial fibrillation; PS, propensity score. ^a^Incidence rate per 100 person-years at risk. ^b^*P* < 0.05

For the ITT analysis, incidence (per 100 person-years) of stroke or systemic embolism was significantly lower with rivaroxaban versus warfarin (2.0 vs. 4.1; hazard ratio [HR], 0.74 [95% CI 0.60–0.91]; *P* = 0.004; Fig. 1). Stroke was more common and contributed to the difference between treatments (1.9 vs. 3.9; HR, 0.74 [95% CI 0.60–0.91]; *P* = 0.005), while systemic embolism was less common and not significantly different between treatments (0.1 vs. 0.3; HR, 0.85 [95% CI 0.36–1.99]; *P* = 0.706). Ischemic stroke was significantly reduced with rivaroxaban versus warfarin (1.8 vs. 3.3; HR, 0.77 [95% CI 0.61–0.96]; *P* = 0.019). The incidence of major bleeding was not significantly different with rivaroxaban versus warfarin (3.8 vs. 6.1; HR, 0.94 [95% CI 0.80–1.10]; *P* = 0.419). Similar nonsignificant findings were observed for intracranial hemorrhage (0.3 vs. 0.8) and major extracranial bleeding (3.7 vs. 5.9; Fig. 1).

On-treatment analysis results were consistent with the ITT analysis, showing a significant reduction in stroke or systemic embolism with rivaroxaban versus warfarin (2.1 vs. 4.8; HR, 0.70 [95% CI 0.55–0.89]; *P* = 0.004; Fig. 1). Incidence rates of stroke (2.0 vs. 4.6) and ischemic stroke (1.9 vs. 4.0) also were significantly reduced with rivaroxaban versus warfarin. Incidence rates were not statistically significant with rivaroxaban versus warfarin for major bleeding (4.0 vs. 7.0; HR, 0.89 [95% CI 0.74–1.08]; *P* = 0.240) or intracranial hemorrhage (0.3 vs. 0.9) and major extracranial bleeding (3.9 vs. 6.8).

### HRU and economic outcomes

Based on the ITT analyses, all-cause HCRU was significantly (*P* < 0.001) reduced with rivaroxaban versus warfarin across all events for the number of events PPPY: inpatient hospitalizations (5.2 vs. 5.6), ED visits (0.5 vs. 0.6), outpatient visits (37.2 vs. 46.5), and pharmacy fills (35.6 vs. 36.0; Table 2). The mean length of hospital stay was significantly shorter with rivaroxaban versus warfarin (9.6 vs. 10.2 days; *P* < 0.0001). Among patients with ≥ 1 hospitalization, the mean length of hospital stay was 12.3 days with rivaroxaban and 12.6 days with warfarin (*P* = 0.0003).

**Table 2:** All-cause healthcare resource utilization for patients with NVAF and sleep apnea (PS overlap weighted results)

	Rivaroxaban (n = 5632)	Warfarin (n = 5632)	Estimate^a^ (95% CI)	*P-*value
ITT analysis				
Patients with ≥ 1 event of interest (%)				
Inpatient hospitalizations	67.2%	71.3%	0.94 (0.95, 0.98)	0.0010
ED visits	43.7%	45.0%	0.97 (0.91, 1.03)	0.3188
Outpatient visits^b^	98.9%	99.2%	1.00 (0.99, 1.00)	0.1810
Pharmacy fills	99.9%	100.0%	1.00 (1.00, 1.00)	0.9523
Number of events of interest (PPPY), mean (SD)				
Inpatient hospitalizations	5.2 (13.2)	5.6 (12.2)	0.93 (0.92, 0.94)	< 0.0001
ED visits	0.5 (1.0)	0.6 (1.2)	0.85 (0.81, 0.89)	< 0.0001
Outpatient visits^b^	37.2 (26.9)	46.5 (30.8)	0.80 (0.79, 0.80)	< 0.0001
Pharmacy fills	35.6 (19.0)	36.0 (19.0)	0.99 (0.98, 1.00)	0.0011
Length of hospital stay in days (PPPY), mean (SD)				
All patients	9.6 (28.8)	10.2 (24.3)	0.94 (0.93, 0.95)	< 0.0001
Patients with ≥ 1 hospitalization	12.3 (32.1)	12.6 (26.4)	0.98 (0.97, 0.99)	0.0003
On-treatment analysis				
Patients with ≥ 1 event of interest (%)				
Inpatient hospitalizations	61.5%	65.2%	0.94 (0.91, 0.98)	0.0051
ED visits	34.8%	35.5%	0.98 (0.91, 1.05)	0.5818
Outpatient visits^b^	98.5%	99.0%	1.00 (0.99, 1.00)	0.1254
Pharmacy fills	99.9%	99.9%	1.00 (1.00, 1.00)	0.9523
Number of events of interest (PPPY), mean (SD)				
Inpatient hospitalizations	5.1 (14.8)	5.8 (14.4)	0.88 (0.86, 0.89)	< 0.0001
ED visits	0.5 (1.1)	0.6 (1.3)	0.82 (0.78, 0.87)	< 0.0001
Outpatient visits^b^	36.1 (27.4)	49.4 (33.4)	0.73 (0.73, 0.74)	< 0.0001
Pharmacy fills	36.0 (19.7)	37.0 (20.5)	0.97 (0.97, 0.98)	< 0.0001
Length of hospital stay in days (PPPY), mean (SD)				
All patients	9.3 (32.7)	10.4 (29.2)	0.89 (0.88, 0.90)	< 0.0001
Patients with ≥ 1 hospitalization	12.3 (34.7)	13.0 (28.0)	0.94 (0.92, 0.95)	< 0.0001

*CI* confidence interval, *ED* emergency department, *ITT* intent-to-treat, *NVAF* nonvalvular atrial fibrillation, *PPPY* per patient per year, *PS* propensity score, *SD* standard deviation

^a^Odds ratio was used to compare the proportion of patients with ≥ 1 event of interest. Rate ratio was used to compare the number of events (PPPY). The difference in means was used to compare the length of stay. Statistical comparisons are comparing rivaroxaban vs. warfarin (reference group)

^b^Outpatient visits included hospital outpatient, physician office, and skilled nursing facility visits

Similar results were obtained for the on-treatment analysis. Rivaroxaban was associated with significantly fewer HCRU events PPPY, with the largest difference for outpatient visits (36.1 vs. 49.4; risk ratio 0.73 [95% CI 0.73–0.74]; Table 2). The mean length of hospital stay was shorter with rivaroxaban versus warfarin for all patients (9.3 vs. 10.4 days) and those with ≥ 1 hospitalization (12.3 vs. 13.0 days).

**Fig. 2:**
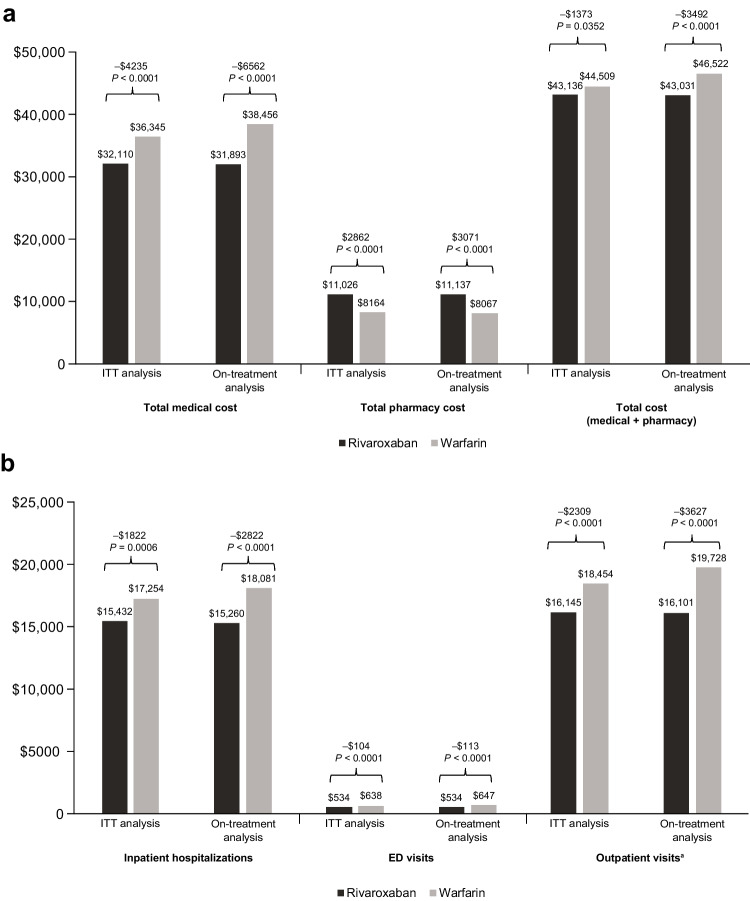
All-cause total healthcare costs (medical + pharmacy) **a** and total medical costs breakdown for NVAF patients with sleep apnea **b**. ED, emergency department; ITT, intent-to-treat; NVAF, nonvalvular atrial fibrillation. ^a^Outpatient visits included hospital outpatient visits, physician office (primary and specialty) visits, and skilled nursing facility visits

Reduced HCRU with rivaroxaban led to significantly (*P* < 0.05) lower total healthcare costs compared with warfarin in both the ITT and on-treatment analyses (Fig. 2a). Total medical cost differences between rivaroxaban and warfarin were −$4235 in the ITT analysis and − $6562 in the on-treatment analysis. Total pharmacy costs were significantly higher with rivaroxaban versus warfarin in the ITT ($2862) and on-treatment ($3071) analyses. Altogether, total healthcare (medical + pharmacy) costs were significantly lower with rivaroxaban versus warfarin (− $1373 in ITT and − $3492 in on-treatment analyses). Among total medical costs, costs for inpatient hospitalizations, ED visits, and outpatient visits were significantly lower with rivaroxaban in both analyses (Fig. 2b). Reduced outpatient visits contributed to the largest cost savings with rivaroxaban versus warfarin in the ITT and on-treatment analyses (− $2309 vs. − $3627, respectively).

### Exploratory analyses

Rivaroxaban was consistently associated with reduced incidence rates of stroke or systemic embolism versus warfarin across patient subgroups in the ITT analysis, with significant reductions in patients < 65 years of age, males, nonobese patients, diabetic patients, patients without heart failure, patients with a history of prior stroke, and those with metabolic syndrome (Online Resource 1; Supplementary Table 1). Owing to the small percentage of patients with confirmed PAP therapy, this subgroup analysis was not performed. Results were similar in the on-treatment analysis except for a significant reduction in stroke/systemic embolism for patients who did not have diabetes and a nonsignificant reduction among patients with diabetes (Online Resource 1; Supplementary Table 2). Consistent with the overall population in both ITT and on-treatment analyses, incidence rates of major bleeding with rivaroxaban versus warfarin were not significantly different across patient subgroups.

NVAF patients with OSA receiving rivaroxaban were significantly more adherent versus those receiving warfarin (49.6% vs. 43.8%; odds ratio, 1.13 [95% CI 1.07–1.20]; *P* < 0.0001).

## Discussion

In this study, rivaroxaban was associated with a reduced risk of stroke/systemic embolism (26% and 30% risk reduction in the ITT and on-treatment follow-up groups, respectively) versus warfarin. Rates of major bleeding were not significantly different with rivaroxaban versus warfarin. The effectiveness of rivaroxaban compared with warfarin was consistent across subgroups. Overall, HCRU and costs for inpatient hospitalizations, ED visits, and outpatient visits were significantly lower in patients receiving rivaroxaban versus warfarin. Lower total medical and total healthcare costs were observed with rivaroxaban over warfarin despite higher pharmacy costs with rivaroxaban.

In a prior study using Optum^®^ electronic health record data, incidence rates of stroke or systemic embolism were similar in patients with NVAF and OSA receiving rivaroxaban and warfarin (0.74% vs. 0.81%; HR, 0.92 [95% CI 0.82–1.03]) [19]. Bleeding-related hospitalization rates were significantly reduced with rivaroxaban compared with warfarin (1.52% vs. 1.81% per year; HR, 0.85 [95% CI 0.78–0.92]) [19]. When the study population was restricted to patients with a moderate-to-high risk of stroke or systemic embolism based on CHA_2_DS_2_-VASc score (≥ 2 in men, ≥ 3 in women), rivaroxaban was associated with significant reductions in the risk of stroke/systemic embolism (HR, 0.67 [95% CI 0.60–0.74]) and bleeding-related hospitalizations (HR, 0.57 [95% CI 0.53–0.62]) [19]. These findings are consistent with the results from the current study among patients with a moderate-to-high risk of stroke defined by CHA_2_DS_2_-VASc score [3].

We observed OSA in 22% of patients with NVAF and moderate-to-severe risk of stroke and nearly 60% of patients with NVAF and OSA had obesity as an additional comorbidity. These conditions are likely bidirectional in cause and effect. A recent real-world evidence study found that 26% of obese patients with NVAF had sleep apnea [20]. In the same study, rivaroxaban reduced the risk of stroke and systemic embolism by 17% and major bleeding by 18%.

The incidence rates of stroke/systemic embolism (per 100 person-years) in our study cohort were higher for rivaroxaban and warfarin (2.0 vs. 4.1) compared with those observed in the phase 3 ROCKET-AF trial of patients with NVAF (1.7 vs. 2.2, respectively) [21], in which 4.53% (645/14,236) of patients had comorbid OSA and 2.26% (322/14,236) were using CPAP. This finding suggests that comorbid OSA in patients with NVAF may increase the risk of thrombotic events.

In the ORBIT-AF registry of 10,132 patients with AF, 18% had comorbid OSA, and these patients had a higher incidence of severe or disabling symptoms, were more often on rhythm-control therapy, and had a higher risk of hospitalization (HR, 1.12 [95% CI 1.03–1.22]) [12]. However, rates of mortality, AF progression, and major cardiovascular events (cardiovascular death, myocardial infarction, and stroke/transient ischemic attack) were similar between patients with and without OSA. In a retrospective analysis of ORBIT-AF I and ORBIT-AF II that included 22,760 patients with AF, approximately 18% of patients had OSA, and those with OSA had an increased risk of major adverse cardiac and neurologic events (cardiovascular death, myocardial infarction, stroke/transient ischemic attack/non-central nervous system systemic embolism, and new-onset heart failure) versus patients without OSA (adjusted HR, 1.16 [95% CI 1.03–1.31]) [22]. In the same study, OSA was an independent risk factor for stroke/systemic embolism beyond CHA_2_DS_2_-VASc risk factors (adjusted HR, 1.38 [95% CI 1.12–1.70]) [22]. A recent retrospective analysis using the National Inpatient Sample database from 2016 to 2017 found OSA in 15% of patients with primary AF hospitalization (*n* = 156,521), and patients with AF and OSA were younger, had more comorbidities, and had received more long-term anticoagulation and inpatient cardioversion compared with those without OSA [23]. However, outcomes of inpatient mortality, cardiovascular events (e.g., cardiac arrest, stroke), major bleeding, and length of stay were similar between the groups with and without OSA [23]. Among 363 patients with AF and OSA in the European Sleep Apnea Database, intermittent hypoxia measured by 4% oxygen desaturation index was significantly associated with cardioembolic risk based on CHA_2_DS_2_-VASc score [24], suggesting a role for OSA in complications like stroke. In a retrospective study of patients undergoing a sleep study, 332 patients had AF, and of these patients, 283 (85.2%) had OSA [25]. Ischemic stroke was more common in patients with OSA versus those without OSA (25.4% vs. 8.2%, respectively; *P* = 0.006), which remained statistically significant after controlling for baseline characteristics significantly associated with stroke (adjusted OR, 3.65 [95% CI 1.252, 10.623]).

### Strengths and study limitations

The IQVIA PharMetrics^®^ Plus claims database is a large database that closely represents the real-world performance of rivaroxaban and warfarin. This retrospective study required a minimum of 12 months of continuous health plan enrollment, which allowed for a better understanding of patient characteristics and longitudinal evaluation of outcomes. The study was limited by possible coding errors and inconsistencies that may occur in administrative claims data. Of note, the presence of sleep apnea testing and treatment at baseline was low, which may be due to baseline characteristics being captured during the 12 months before the index date rather than the full medical history. Despite efforts to balance the study cohorts, residual confounding cannot be excluded. Results may not be generalizable to the entire US population, and conclusions are limited to the studied population that included younger patients with primarily commercial or self-insurance. The claims data do not allow an assessment of OSA severity to determine its effect on the outcomes. The presence of a claim for prescription medication does not indicate that the medication was taken as prescribed, and medications that are commonly available over the counter, such as aspirin, would be under-reported. Because laboratory data are limited in the database, time in the therapeutic international normalized ratio range was not assessed for patients using warfarin.

## Conclusions

Among patients with NVAF and comorbid OSA who were at moderate-to-high risk of stroke, rivaroxaban was associated with a significant reduction in stroke/systemic embolism with a similar risk of major bleeding compared with warfarin. HCRU and total healthcare costs were significantly reduced in patients receiving rivaroxaban versus those receiving warfarin. Taken together, this analysis provides real-world evidence supporting the use of rivaroxaban in patients with NVAF and OSA.

## Supplementary Information

Below is the link to the electronic supplementary material.

Supplementary file1 (DOCX 236 KB)

## Data Availability

The data sharing policy of Janssen Pharmaceutical Companies of Johnson & Johnson is available at https://www.janssen.com/clinical-trials/transparency. These data were made available by IQVIA and used under license for the current study and are not publicly available.
