# Supplementary material for: Clinical and economic outcomes with rivaroxaban versus warfarin in patients with nonvalvular atrial fibrillation and obstructive sleep apnea: retrospective analysis of US healthcare claims
Source: J Interv Card Electrophysiol. 2024 Nov 25;68(3):613–24. doi: 10.1007/s10840-024-01940-6 (PMC12167291; doi:10.1007/s10840-024-01940-6)
Supplement: Supplementary file 1 — Supplementary file1 (DOCX 236 KB) [file 10840_2024_1940_MOESM1_ESM.docx]

**Online Resource 1**

**Supplementary Fig 1 Study design**

**
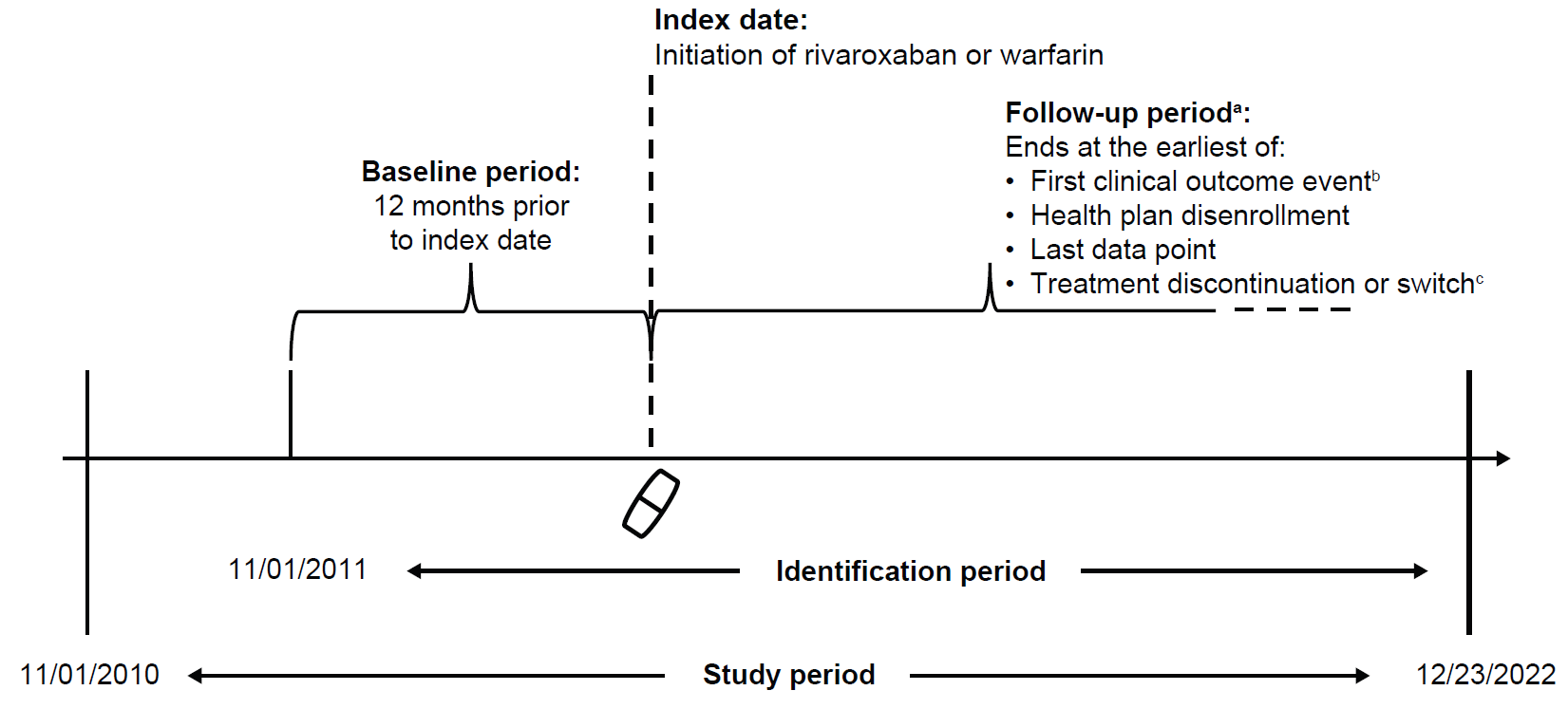
**

^a^Two follow-up approaches will be used to evaluate clinical outcomes: on-treatment and intention-to-treat.

^b^For effectiveness: first stroke or systemic embolism; for safety: first major bleeding.

^c^On-treatment analysis also includes discontinuation of index treatment with allowable gap of 60 days between fills or a treatment change before the gap.

**Supplementary Fig 2 Patient attrition**

**
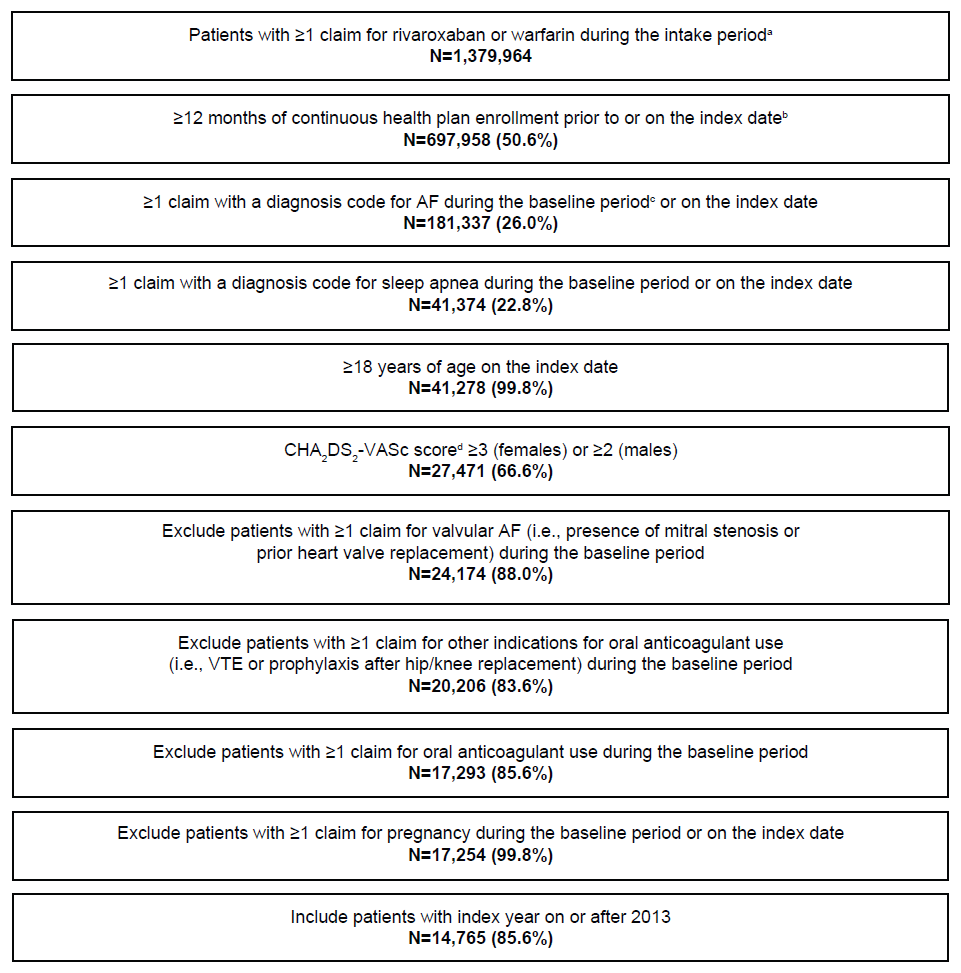
**

AF, atrial fibrillation; VTE, venous thromboembolism.

^a^Intake period defined as November 1, 2011, through December 23, 2022.

^b^Index date defined as the fill date of the first rivaroxaban or warfarin claim.

^c^Baseline period defined as 12 months prior to the index date.

^d^CHA_2_DS_2_-VASc score was calculated during baseline period.

**Supplementary Table 1. Outcomes across subgroups for patients with NVAF and sleep apnea (weighted results; ITT analysis)**

|  | **Effectiveness (stroke or systemic embolism)** | | | | **Major bleeding** | | | |
| --- | --- | --- | --- | --- | --- | --- | --- | --- |
|  | **Rivaroxaban**  **IR^a^  (95% CI)** | **Warfarin**  **IR^a^  (95% CI)** | **HR (95% CI)** | ***P*-value** | **Rivaroxaban**  **IR^a^  (95% CI)** | **Warfarin**  **IR^a^  (95% CI)** | **HR (95% CI)** | ***P*-value** |
| **Age** |  |  |  |  |  |  |  |  |
| Age ≥65 years (n=6426) | 2.36  (2.04, 2.72) | 3.68  (3.22, 4.18) | 0.93  (0.69, 1.25) | 0.6180 | 5.14  (4.66, 5.67) | 6.77  (6.13, 7.46) | 1.02  (0.82, 1.26) | 0.8958 |
| Age <65 years (n=8339) | 1.80  (1.57, 2.06) | 4.47  (3.94, 5.05) | 0.61  (0.46, 0.82) | 0.0010 | 2.84  (2.54, 3.16) | 5.44  (4.86, 6.08) | 0.87  (0.68, 1.11) | 0.2554 |
| **Sex** |  |  |  |  |  |  |  |  |
| Male (n=11,057) | 1.90  (1.69, 2.31) | 3.85  (3.45, 4.28) | 0.72  (0.56, 0.92) | 0.0087 | 3.43  (3.14, 3.74) | 5.80  (5.31, 6.33) | 0.94  (0.77, 1.14) | 0.4963 |
| Female (n=3708) | 2.46  (2.03, 2.95) | 4.64  (3.92, 5.45) | 0.82  (0.56, 1.20) | 0.3022 | 4.86  (4.25, 5.54) | 7.03  (6.13, 8.03) | 0.93  (0.69, 1.26) | 0.6380 |
| **Obesity** |  |  |  |  |  |  |  |  |
| Obese (n=8606) | 2.00  (1.75, 2.28) | 3.94  (2.02, 2.58) | 0.77  (0.58, 1.02) | 0.0703 | 4.04  (3.67, 4.43) | 6.34  (5.74, 6.98) | 0.96  (0.78, 1.19) | 0.7296 |
| Nonobese (n=6159) | 2.07  (1.78, 2.39) | 4.19  (3.67, 4.77) | 0.73  (0.54, 0.98) | 0.0391 | 3.41  (3.04, 3.82) | 5.86  (5.23, 6.55) | 0.92  (0.71, 1.18) | 0.5022 |
| **Diabetes** |  |  |  |  |  |  |  |  |
| Yes (n=8596) | 1.96  (1.70, 2.24) | 4.16  (3.70, 4.66) | 0.74  (0.57, 0.98) | 0.0359 | 3.99  (3.62, 4.39) | 6.72  (6.12, 7.36) | 0.95  (0.77, 1.17) | 0.6357 |
| No (n=6169) | 2.13  (1.84, 2.45) | 3.90  (3.36, 4.49) | 0.74  (0.54, 1.01) | 0.0606 | 3.49  (3.12, 3.90) | 5.26  (4.64, 5.95) | 0.90  (0.70, 1.17) | 0.4474 |
| **Heart failure** |  |  |  |  |  |  |  |  |
| Yes (n=6870) | 2.41  (2.07, 2.78) | 4.56  (4.04, 5.14) | 0.79  (0.60, 1.06) | 0.1166 | 5.30  (4.79, 5.85) | 8.48  (7.74, 9.27) | 0.93  (0.75, 1.15) | 0.4829 |
| No (n=7895) | 1.80  (1.57, 2.05) | 3.56  (3.10, 4.06) | 0.70  (0.52, 0.94) | 0.0189 | 2.83  (2.55, 3.15) | 3.92  (3.44, 4.45) | 0.93  (0.72, 1.21) | 0.5998 |
| **Prior stroke** |  |  |  |  |  |  |  |  |
| Yes (n=2033) | 7.36  (6.24, 8.64) | 14.90  (13.13, 16.84) | 0.59  (0.43, 0.81) | 0.0013 | 4.49  (3.66, 5.46) | 7.37  (6.24, 8.65) | 0.82  (0.55, 1.24) | 0.3502 |
| No (n=12,732) | 1.43  (1.27, 1.62) | 2.25  (1.97, 2.56) | 0.89  (0.68, 1.18) | 0.4163 | 3.67  (3.39, 3.97) | 5.86  (5.39, 6.37) | 0.95  (0.80, 1.14) | 0.5755 |
| **Metabolic syndrome** |  |  |  |  |  |  |  |  |
| Yes (n=9890) | 2.04  (1.81, 2.31) | 4.26  (3.82, 4.73) | 0.73  (0.56, 0.94) | 0.0136 | 4.02  (3.68, 4.39) | 6.67  (6.11, 7.28) | 0.94  (0.77, 1.14) | 0.5323 |
| No (n=4875) | 2.01  (1.70, 2.37) | 3.65  (3.09, 4.29) | 0.77  (0.54, 1.11) | 0.1587 | 3.29  (2.88, 3.75) | 5.06  (4.38, 5.80) | 0.93  (0.69, 1.25) | 0.6257 |

CI, confidence interval; HR, hazard ratio; IR, incidence rate; ITT, intent-to-treat; NVAF, nonvalvular atrial fibrillation.

^a^Per 100 person-years at risk.

**Supplementary Table 2. Outcomes across subgroups for patients with NVAF and sleep apnea (weighted results; on-treatment analysis)**

|  | **Effectiveness (stroke or systemic embolism)** | | | | **Major bleeding** | | | |
| --- | --- | --- | --- | --- | --- | --- | --- | --- |
|  | **Rivaroxaban**  **IR^a^  (95% CI)** | **Warfarin**  **IR^a^  (95% CI)** | **HR (95% CI)** | ***P* value** | **Rivaroxaban**  **IR^a^  (95% CI)** | **Warfarin**  **IR^a^  (95% CI)** | **HR (95% CI)** | ***P* value** |
| **Age** |  |  |  |  |  |  |  |  |
| Age ≥65 years (n=6426) | 2.46  (2.05, 2.91) | 4.23  (3.61, 4.92) | 0.92  (0.65, 1.31) | 0.6358 | 3.62  (3.12, 4.18) | 7.71  (6.86, 8.65) | 0.97  (0.75, 1.26) | 0.8012 |
| Age <65 years (n=8339) | 1.94  (1.65, 2.26) | 5.46  (4.74, 6.26) | 0.57  (0.41, 0.79) | 0.0009 | 2.96  (2.61, 3.36) | 6.33  (5.56, 7.19) | 0.83  (0.63, 1.11) | 0.2178 |
| **Sex** |  |  |  |  |  |  |  |  |
| Male (n=11,057) | 1.88  (1.63, 2.16) | 4.53  (4.00, 5.12) | 0.63  (0.47, 0.84) | 0.0018 | 3.52  (3.17, 3.90) | 6.74  (6.08, 7.45) | 0.85  (0.68, 1.07) | 0.1759 |
| Female (n=3708) | 3.06  (2.47, 3.75) | 5.70  (4.70, 6.86) | 0.93  (0.60, 1.43) | 0.7258 | 5.49  (4.68, 6.40) | 7.85  (6.66, 9.20) | 0.99  (0.69, 1.41) | 0.9345 |
| **Obesity** |  |  |  |  |  |  |  |  |
| Obese (n=8606) | 2.10  (1.79, 2.45) | 4.71  (3.89, 5.17) | 0.73  (0.53, 1.02) | 0.0657 | 4.29  (3.84, 4.78) | 7.21  (6.42, 8.07) | 0.95  (0.74, 1.22) | 0.6777 |
| Nonobese (n=6159) | 2.19  (1.83, 2.60) | 4.98  (4.27, 5.78) | 0.70  (0.49, 1.00) | 0.0485 | 3.52  (3.05, 4.03) | 6.80  (5.95, 7.73) | 0.84  (0.62, 1.14) | 0.2593 |
| **Diabetes** |  |  |  |  |  |  |  |  |
| Yes (n=8596) | 2.00  (1.69, 2.35) | 4.72  (4.12, 5.39) | 0.74  (0.53, 1.03) | 0.0728 | 4.17  (3.72, 4.67) | 8.08  (7.27, 8.96) | 0.84  (0.66, 1.08) | 0.1688 |
| No (n=6169) | 2.30  (1.94, 2.71) | 5.00  (4.24, 5.85) | 0.67  (0.47, 0.95) | 0.0256 | 3.70  (3.23, 4.21) | 5.49  (4.69, 6.38) | 0.96  (0.70, 1.31) | 0.7803 |
| **Heart failure** |  |  |  |  |  |  |  |  |
| Yes (n=6870) | 2.48  (2.08, 2.94) | 5.25  (4.55, 6.02) | 0.77  (0.55, 1.08) | 0.1313 | 5.56  (4.94, 6.24) | 9.92  (8.94, 10.99) | 0.86  (0.67, 1.10) | 0.2366 |
| No (n=7895) | 1.92  (1.64, 2.24) | 4.41  (3.77, 5.13) | 0.65  (0.46, 0.92) | 0.0142 | 2.95  (2.59, 3.34) | 4.22  (3.59, 4.92) | 0.93  (0.68, 1.28) | 0.6651 |
| **Prior stroke** |  |  |  |  |  |  |  |  |
| Yes (n=2033) | 9.67  (8.11, 11.46) | 20.91  (18.30, 23.78) | 0.58  (0.41, 0.81) | 0.0016 | 4.64  (3.61, 5.87) | 8.50  (6.98, 10.25) | 0.79  (0.49, 1.28) | 0.3375 |
| No (n=12,732) | 1.29  (1.10, 1.51) | 2.14  (1.80, 2.52) | 0.90  (0.63, 1.28) | 0.5624 | 3.87  (3.53, 4.24) | 6.72  (6.10, 7.40) | 0.91  (0.74, 1.12) | 0.3584 |
| **Metabolic syndrome** |  |  |  |  |  |  |  |  |
| Yes (n=9890) | 2.08  (1.79, 2.41) | 4.92  (4.33, 5.56) | 0.69  (0.51, 0.94) | 0.0175 | 4.22  (3.80, 4.67) | 7.76  (7.00, 8.57) | 0.87  (0.69, 1.10) | 0.2496 |
| No (n=4875) | 2.24  (1.84, 2.70) | 4.67  (3.88, 5.58) | 0.73  (0.49, 1.09) | 0.1272 | 3.48  (2.98, 4.06) | 5.60  (4.72, 6.59) | 0.92  (0.65, 1.31) | 0.6497 |

CI, confidence interval; HR, hazard ratio; IR, incidence rate; NVAF, nonvalvular atrial fibrillation.

^a^Per 100 person-years at risk.
